# Immune Resilience: Rewriting the Rules of Healthy Aging

**DOI:** 10.1111/acel.70089

**Published:** 2025-04-30

**Authors:** Monty Montano

**Affiliations:** ^1^ Harvard Medical School Boston Massachusetts USA

**Keywords:** aging, centenarians, molecular biology of aging, senescence

## Abstract

Aging is typically framed by disease, not resilience. This Perspective highlights immune resilience (IR) as a core determinant of healthy aging, based on new findings linking TCF7‐driven immune profiles to extended healthspan and lifespan. IR buffers against immunosenescence, inflammaging, and senescent cell phenotypes, with benefits most pronounced before age 70. By reframing aging around salutogenesis rather than pathogenesis, this work shifts the focus toward resilience mechanisms and composite traits preserving health.

## Graphical abstract

Three forces influencing the aging process: salutogenesis, pathogenesis, and the biology of aging (senescence).

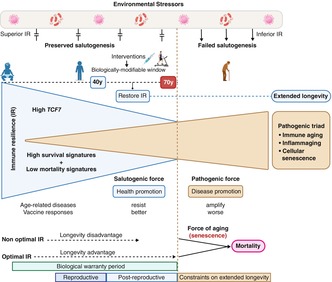

Salutogenesis—from the Latin *salus* (health) and Greek *genesis* (origin)—reorients resilience and aging research by shifting the central question from “What causes disease?” to “How do people actively sustain health in the face of adversity?” First proposed by Antonovsky (1980), this framework remains overshadowed by pathology‐focused approaches. PubMed data reveal a striking 2400:1 ratio imbalance, with 10.8 million studies on pathogenesis versus just 4473 on salutogenesis. This disparity permeates related fields. For example, while there are 1 million papers on inflammation and 64,000 on “healthy aging,” the overwhelming focus remains on deficits and disease mechanisms rather than on how health is actively maintained and promoted.

Three pivotal questions emerge: Why does scientific inquiry favor disease management over health cultivation? What biological mechanisms enable proactive health preservation? How can researchers and funders pivot their systems to prioritize salutogenesis?

In this issue of *Aging Cell*, Sunil Ahuja's team pioneers a unified model linking immune resilience (IR)—the dual capacity to defend against pathogens and regulate inflammation—with salutogenesis (Manoharan et al. 2025). Their data‐rich study, “The 15‐Year Survival Advantage: Immune Resilience as a Salutogenic Force in Healthy Aging,” reframes aging as a lifelong tug‐of‐war among three forces: (1) health‐preserving IR mechanisms (salutogenesis), (2) disease‐driving processes (pathogenesis), and (3) irreversible functional decline (senescence) (Figure 1).

**FIGURE 1:**
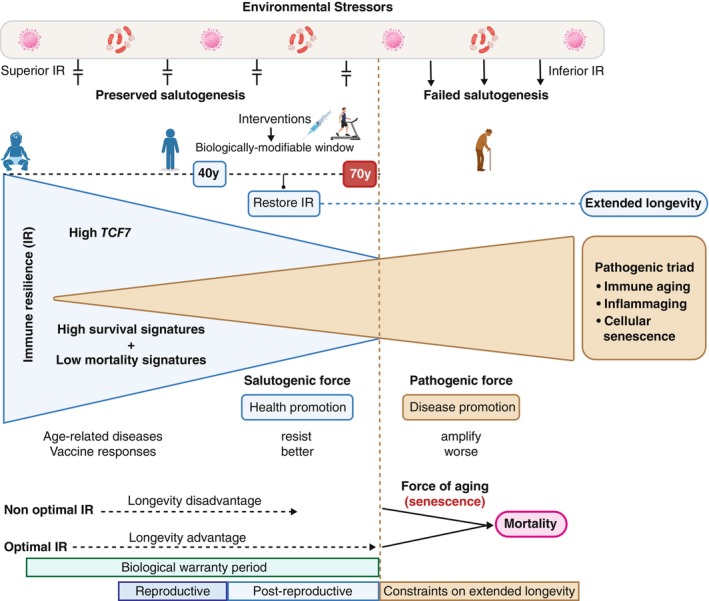
Three forces influencing the aging process: Salutogenesis, pathogenesis and the biology of aging. Diagram courtesy of Kian Andampour.

Crucially, the authors distinguish salutogenesis from being merely the absence of pathogenesis. While pathogenesis asks “What causes disease?”, salutogenesis probes “What sustains health?” They show that optimal IR functions as a salutogenic buffer to counter key drivers of age‐related diseases, infections, and mortality, which they term the “pathogenic triad”: immune aging (immunosenescence), inflammaging (chronic inflammation of aging), and senescent cell accumulation. Centenarians exemplify this salutogenic principle, often escaping age‐related conditions entirely. For example, Lucile Randon, a French nun who developed COVID‐19 days before her 117th birthday, but remained asymptomatic, becoming the oldest known survivor. She died in her sleep on January 17, 2023, at the age of 118 years.

In their salutogenic framework, optimal IR—characterized by elevated T‐cell factor 7 (*TCF7*) and a core repertoire of immune‐enhancing transcription factors—maximizes responses to environmental stressors while minimizing collateral damage from the pathogenic triad. This constellation functions as a multidimensional protective trait, extending both healthspan and lifespan. By quantifying these processes through a suite of gene expression signatures and other IR metrics (Manoharan et al. 2025; Ahuja et al. 2023), they demonstrate that IR‐associated salutogenesis tracks with lower levels of the pathogenic triad, potentially explaining its association with better outcomes for infectious diseases, vaccination, and non‐infectious conditions such as cardiovascular disease and dementia—as well as superior immune and inflammatory biomarker profiles, regardless of age. This sophisticated evidence‐based framework recalibrates our understanding of inflammatory stress responses and their impact on health trajectories, independent of age.

Synthesizing four decades of research from pioneers such as Olshansky, Finch, Hayflick, and Vaupel, the work identifies two critical windows for IR effects: a “biological warranty period” (during which IR optimization could extend healthy lifespan by 15 years) and a ~70‐year threshold beyond which IR's protective capacity wanes. This paradigm urges a shift in clinical practice—from managing decline to preemptively boosting IR—offering time‐sensitive strategies to prolong vitality before irreversible senescence dominates.

## 1 IR Trajectories Driving Healthspan and Lifespan

From an evolutionary standpoint, acute inflammation is essential for survival, but its dysregulation with age fuels chronic disease. The study categorizes IR trajectories during inflammatory stress into three groups: (1) IR‐preservers (maintain optimal IR under stress, exhibit minimal pathogenic triad burden, and display robust health benefits), (2) IR‐reconstituters (transiently disrupted IR with post‐stress recovery), and (3) IR‐degraders (persistent IR deterioration and heightened triad burden, with worse outcomes). This stratification equips clinicians with actionable insights for risk assessment and personalized interventions.

## 2 IR As a Cross‐Cutting Determinant of Healthspan and Lifespan

The manuscript gains strength from its multi‐cohort validation, analyzing over 17,500 individuals across age groups, populations, and inflammatory stressors, including the Framingham Heart Study and the Vitality 90+ cohort. This breadth enables rigorous assessment of IR responses to diverse stressors—ranging from infections (e.g., RSV, COVID‐19, tuberculosis) and vaccines to myocardial ischemia and controlled human challenge trials (e.g., influenza, Salmonella). By demonstrating IR's systemic role in contexts from cardiovascular disease to Alzheimer's disease (AD)—and correlating with brain health and other biomarkers—the work positions IR as a universal determinant of aging trajectories. Four clinical vignettes underscore this universality.

Vignette 1. Optimal IR confers a 69% reduction in mortality in the Framingham and COVID‐19 cohorts, with the strongest effects seen in individuals under age 70. Strikingly, 40‐year‐old IR‐degraders face a sex‐adjusted hazard ratio exceeding 9.0 compared to their optimal‐IR peers—equivalent to the mortality risk of 55‐year‐old optimal‐IR individuals, indicating a 15‐year survival advantage. Optimal IR also correlates with reduced cardiovascular incidents after adjusting for traditional risk factors.

Vignette 2. Analysis of the Vitality 90+ cohort reveals that even among nonagenarians and centenarians, those with impaired salutogenesis exhibit greater triad burden and elevated cell‐free DNA levels—with corresponding increases in mortality risk. Notably, even among centenarians, those with preserved IR exhibit lower cell‐free DNA levels. This approach avoids the ecological fallacy by directly linking individual‐level biomarkers to outcomes.

Vignette 3. Examination of Alzheimer's disease (AD) cohorts uncovers three critical insights that challenge age‐centric models. First, IR impairment is more common in AD patients than in age‐matched controls and those with mild cognitive impairment. Second, while AD patients show higher triad burden than controls, this difference attenuates with advancing age, suggesting mechanisms independent of chronological aging. Third, patients maintaining optimal IR markers demonstrate reduced pathogenic triad burden, highlighting IR's protective role in neurodegeneration.

Vignette 4. The study presents compelling data showing that maintaining optimal IR correlates with robust cross‐pathogen defense. During COVID‐19, extreme IR‐degraders faced adverse events across three phases: pre‐COVID‐19 (higher pre‐existing comorbidities), acute infection (higher hospitalization/mortality risks), and the post‐acute phase (persistent mortality). Conversely, IR‐preservers manifested near‐complete protection against hospitalization and mortality, with superior immune profiles regardless of age.

## 3 IR Balances Immune‐Regenerative vs. Inflammatory Molecular Pathways

Beyond observational insights, the manuscript points to actionable pathways, highlighting a core IR biomarker signature: individuals with impaired IR show higher levels of pro‐inflammatory factors like IL‐6 and IGFBP2, while those with optimal IR display markers of immune strength such as CD23 alongside tissue and growth‐supportive proteins like epidermal growth factor receptor, soluble receptor for IGF‐1 (insulin‐like growth factor 1) and IGFBP‐7, with the IGF system playing a fundamental role in the aging process. These biomarker data establish IR as a key link between the IGF system and immune health, proposing that optimal IR may have evolved as a dual‐defense mechanism—simultaneously reducing susceptibility to fatal infections and mitigating chronic metabolic disorders, which are associated with an increased risk of cardiovascular diseases and AD.

TCF7, whose gene product is TCF1, functions via the Wnt signaling pathway, and plays an essential role in the development and maintenance of stemness in T cells (Sturmlechner et al. 2023; Zhao et al. 2022). These findings suggest that a *TCF7* network—a conserved regulator of immune–organ crosstalk—triggers dual pathologies: heightened inflammatory burden and depletion of trophic‐regenerative factors. This perspective moves beyond viewing IR as a simple immune measure, instead framing it as a dynamic interface between immunity and systemic health.

## 4 Aging, Longevity, and the Evolutionary Paradox: Time‐Bound Salutogenesis

Aging remains evolution's great contradiction: natural selection favors reproductive success, yet humans—and a few other species—defy this logic by sustaining a post‐reproductive “warranty period” that spans decades. In contemporary societies, this period has lengthened dramatically due to lower infant mortality, reduced environmental risks, and medical advances—a phenomenon termed “manufactured longevity” by James Vaupel. This lengthening raises a critical question: How have we managed to extend lifespan so radically without fundamentally altering the biological mechanisms of decline?

The study's framework and results address this paradox by separating two mechanistic forces: anabolic systems that build resilience (salutogenic processes that counter chronic stressors such as inflammation) and entropy‐driven catabolic pathways (pathogenic processes that degrade cellular maintenance through mechanisms such as telomere shortening and epigenetic drift). This synthesis clarifies that medical advances extend life primarily by strengthening resilience, not by addressing the core processes of aging. The paradox dissolves when we see longevity gains as a temporary balance—longer survival masks ongoing underlying decline. Early investments in maintenance systems, while beneficial, may paradoxically accelerate late‐life deterioration (entropy), highlighting the complexity of these trade‐offs.

## 5 Sex Differences Illuminate Evolutionary Trade‐Offs

The salutogenic framework becomes even more compelling when considering clear sex differences in aging. Females demonstrate greater salutogenic capacity linked to IR, hinting at evolutionary trade‐offs between reproductive needs—especially the metabolic demands of pregnancy—and lifelong resilience maintenance. Thus, one of the most compelling aspects of this work is its integration of evolutionary theory with population‐level data. The authors propose that IR evolved under dual pressures: to ensure pathogen defense during reproductive years and to support intergenerational survival—particularly among females. This is consistent with the grandmother hypothesis and may underlie the observed female advantage in longevity and immune competence. This sex‐based divergence underscores the framework's explanatory power for population‐level variation in aging patterns—and why one‐size‐fits‐all anti‐aging interventions may falter.

## 6 Therapeutic Implications: Widening the Resilience‐Entropy Gap

By reframing anti‐aging research as a strategic battle to widen the gap between IR and entropy, the salutogenic framework shifts the perspective from the impossible goal of “cheating death” to the achievable target of delaying biological failure—a challenge compounded by evolutionary and sex‐specific constraints. The protective effects of optimal IR are most pronounced before age 70, primarily guarding against premature mortality in the warranty period. After this threshold, age‐related decline in resilience mechanisms reduces these benefits, reflecting evolution's focus on survival through reproductive years rather than extreme longevity. This defines a critical pre‐70 therapeutic window where IR‐enhancing interventions can have maximal impact.

Actionable targets include boosting immune competence and dampening inflammation linked to the IR program, with TNFα inhibitors showing promise in reversing inflammatory aspects of IR. With validated IR metrics, it is now possible to track the impact of lifestyle changes—such as diet and exercise—and to explore whether the broad benefits of widely used drugs such as GLP‐1 agonists extend beyond glycemic control via IR.

## 7 Concluding Comments

By identifying *TCF7* and IR metrics as both biomarkers and potential regulators of resilience, this study reframes the biology of aging. Instead of tracking disease accumulation or biological age acceleration, it calls for the development of resilience‐focused metrics—ones that capture an individual's capacity to resist and recover from physiological stress. “Failed salutogenesis”—at any age—serves as a pathogenic driver that predisposes individuals to adverse outcomes regardless of age. This salutogenic perspective invites a rethinking of clinical aging models, public health strategies, and personalized interventions aimed at sustaining health across the lifespan.

In sum, Ahuja and colleagues provide compelling evidence that immune resilience—anchored in *TCF7* expression—is a measurable, modifiable, and evolutionarily conserved determinant of healthy aging. Their work exemplifies a next‐generation approach to geroscience—one that shifts the aging narrative from inevitable decline to adaptive capacity, and from pathology to possibility.

## Author Contributions

**Monty Montano:** drafted and edited the manuscript.

## Conflicts of Interest

The author declares no conflicts of interest.

## Data Availability

Data sharing is not applicable to this article as no new data were created or analyzed in this study.
